# Protective Effect of Ursolic Acid on the Intestinal Mucosal Barrier in a Rat Model of Liver Fibrosis

**DOI:** 10.3389/fphys.2019.00956

**Published:** 2019-07-30

**Authors:** Wang Zhang, Dakai Gan, Jie Jian, Chenkai Huang, Fangyun Luo, Sizhe Wan, Meichun Jiang, Yipeng Wan, Anjiang Wang, Bimin Li, Xuan Zhu

**Affiliations:** ^1^Department of Gastroenterology, The First Affiliated Hospital of Nanchang University, Nanchang, China; ^2^Department of Liver Disease, The Ninth Hospital of Nanchang, Nanchang, China

**Keywords:** hepatic fibrosis, intestinal mucosal barrier function, ursolic acid, NOX, intestinal microbiota

## Abstract

Oxidative stress mediated by nicotinamide adenine dinucleotide phosphate (NADPH) oxidase (NOX) plays an important role in intestinal mucosal barrier damage in various disease states. Recent evidence suggests that intestinal mucosal barrier damage and intestinal dysbiosis occur in mice with hepatic fibrosis induced by CCl4 or bile duct ligation. Another study showed that ursolic acid (UA) attenuates experimental colitis via its anti-inflammatory and antioxidant activities. The goal of this study was to investigate the effects of UA on the intestinal mucosal barrier in CCl4-induced hepatic fibrosis in rats and identify its associated mechanisms. Male Sprague-Dawley rats were randomly divided into the following 3 groups (*n* = 10/group): the control, CCl4 model and UA treatment groups. Rats were sacrificed at 72 h after the hepatic fibrosis model was established and assessed for liver fibrosis, intestinal injury, enterocyte apoptosis, bacterial translocation, system inflammation, intestinal oxidative stress, and tight junction protein and NOX protein expression. The results demonstrated that UA attenuated the following: (i) liver and intestinal pathological injury; (ii) cleaved caspase-3 expression in the ileal epithelial cells; (iii) serum lipopolysaccharide and procalcitonin levels; (iv) intestinal malondialdehyde levels; and (v) the expression of the NOX protein components NOX2 and P67phox in ileal tissues. Furthermore, our results suggested that UA improved intestinal dysbiosis and the expression of the tight junction proteins Claudin 1 and Occludin in the ileum of rats. These results indicate that UA has protective effects on the intestinal mucosal barrier in rats with CCl4-induced liver fibrosis by inhibiting intestinal NOX-mediated oxidative stress. Our findings may provide a basis for further clinical studies of UA as a novel and adjuvant treatment to cure liver fibrosis.

## Introduction

Liver fibrosis is a wound-healing response to chronic liver injury that develops into liver cirrhosis or liver cancer, which is associated with significant morbidity and mortality. Progressive fibrosis can eventually result in cirrhosis, liver failure or hepatocellular carcinoma. Intestinal mucosal barrier damage and dysbiosis have been reported to occur in mice with hepatic fibrosis induced by CCl4 or bile duct ligation (Fouts et al., 2012). The increased intestinal permeability resulting from intestinal mucosal barrier damage and intestinal dysbiosis contributes to the translocation of bacteria and/or bacterial products (Fouts et al., 2012; Hartmann et al., 2012), the latter of which induce hepatic stellate cell (HSC) activation and contribute to liver fibrosis (Seki et al., 2007; Hartmann et al., 2012). Moreover, translocated bacteria and their products are closely associated with various liver cirrhosis complications. Therefore, restoring intestinal barrier function and preventing bacterial translocation has great significance for inhibiting the progression of liver fibrosis and improving the prognosis of patients with chronic liver disease.

Various factors contribute to intestinal barrier damage, with intestinal oxidative stress having an especially important role. Nicotinamide adenine dinucleotide phosphate (NADPH) oxidase (NOX) is a multicomponent enzyme complex that generates reactive oxygen species (ROS) in response to various stimulus. NOX-derived ROS is one of the most important sources of intestinal oxidative stress. Welak et al. (2014) observed that NOX2-mediated oxidative stress plays a crucial role in the progression of necrotizing enterocolitis. In another study, the activation of the inflammasome by NOX2-derived ROS was shown to promote ileal mucositis induced by irinotecan, a chemotherapeutic agent that inhibits topoisomerase I (Arifa et al., 2014). Taken together, these results indicate that the inhibition of NOX-mediated oxidative stress can protect the intestinal mucosal barrier.

Ursolic acid (UA) is a natural pentacyclic triterpenoid with various pharmacological activities. In our previous studies, we showed that UA has unique anti-fibrotic effects, inhibiting the proliferation of activated HSCs and inducing their apoptosis but not hepatocyte apoptosis (Shen et al., 2008; Gan et al., 2018). Other researchers subsequently corroborated that UA selectively induces the apoptosis of activated HSCs without inducing liver or quiescent HSC apoptosis (Wang et al., 2011). Furthermore, we observed that UA inhibited the leptin-mediated expression of the NOX subunits NOX2, P67phox and NOX4 in an activated rat HSC cell line (HSC-T6), resulting in the accumulation of extracellular matrix (ECM) proteins (Wang et al., 2011; He et al., 2015). In addition, the results of another study suggested that UA attenuates experimental colitis in mice via its anti-inflammatory and antioxidant activities (Chun et al., 2014; Liu et al., 2016). However, because it is unknown whether UA has a protective effect on the intestinal mucosal barrier in rats with CCl4-induced liver fibrosis, the goal of the current study was to answer this question and elucidate the related mechanism.

## Materials and Methods

### Reagents and Antibodies

The following reagents were used in this study: CCl4 and olive oil (Shandong Xiya Chemical Industries Co., Ltd., Shandong, China); UA (Sigma Chemical Co., St. Louis, United States); Picro Sirius Red Stain and triglyceride assay kits (Beijing Solarbio Science & Technology Co., Ltd., Beijing, China); total antioxidant capacity (TAC), malondialdehyde (MDA), total bilirubin (TBIL), hydroxyproline, alanine aminotransferase (ALT) and C-reactive protein (CRP) assay kits (Nanjing Jiancheng Bioengineering Institute, Nanjing, China); a rat tumor necrosis factor alpha (TNF-α) enzyme-linked immunosorbent assay (ELISA) kit, a rat albumin ELISA kit, a rat procalcitonin ELISA kit and a lipopolysaccharide (LPS) ELISA kit (Elabscience Biotechnology Co., Ltd., Wuhan, China); protein lysis buffer and protease inhibitor (Vazyme Biotech Co., Ltd., Nanjing, China); a Bradford Protein Assay kit (Tiangen Biotech Co., Ltd., Beijing, China); an anti-cleaved caspase-3 antibody (Cell Signaling Technology, United States); anti-NOX2/gp91phox, anti-p67phox, anti-collagen I, anti-alpha smooth muscle actin (α-SMA) and anti-Occludin antibodies (Abcam, United Kingdom); an anti-Claudin 1 antibody (Thermo Fisher Scientific, United States); and mouse anti-β-actin and horseradish peroxidase-labeled goat anti-mouse IgG and goat anti-rabbit IgG antibodies (Beijing Zhongshan Golden Bridge Biotechnology, Co., Ltd., Beijing, China).

### Animal Procedures and Treatments

All experimental procedures were approved by the Institutional Animal Care and Use Committee of the First Affiliated Hospital of Nanchang University (Nanchang, China). All animals received humane care in compliance with institutional guidelines. Male Sprague-Dawley rats (160–200 *g* body weight) were obtained from the Department of Laboratory Animal Science of Nanchang University and had access to water and standard chow diet *ad libitum*. Animals were maintained in an environment with a 12:12 h light/dark cycle, a room temperature of 22 ± 2°C, and 55 ± 5% humidity. Male Sprague-Dawley rats were randomly divided into the following 3 groups (*n* = 10/group): the control, CCl4 model and UA treatment groups (CTR, CCl4 and UAT, respectively). The control rats were given olive oil (2 ml/kg) by gavage twice a week for 8 weeks and then were administered normal saline (40 mg/kg/day) for 4 weeks. Hepatic fibrosis was induced by gastric gavage of CCl4 (diluted 1:4 in olive oil, 2 ml/kg) twice a week for 8 weeks, after which the rats in the CCl4 model and UA treatment groups were given normal saline or UA (40 mg/kg/day) for 4 weeks, respectively. Abdominal fur was removed with a depilatory and the skin was sterilized with iodine, then laparotomies were performed under strict aseptic conditions. Blood samples were collected from the inferior vena cava, and rats were sacrificed afterward. Liver and ileal tissues adjacent to the cecum were isolated. A portion of each liver and ileum was removed for histopathological examination by fixation with 10% formalin and subsequent embedding with paraffin. The remaining tissue specimens were frozen in liquid nitrogen and stored at −80°C.

### Liver and Ileum Histopathology

The paraffin-embedded liver and ileum samples were used to prepare 5 μm thick slices with a microtome. The slices were stained with hematoxylin and eosin using standard methods. For Sirius red collagen staining, the liver slices were deparaffinized and stained with Picro Sirius Red for 1 h at room temperature. After washing, the slices on the slides were stained with hematoxylin and subsequently mounted in permount medium. The degree of hepatic fibrosis was evaluated semiquantitatively based on the Metavir score (Fagan et al., 2015). The Sirius red stained area was quantified by Image-Pro Plus 6.0. The histological grade of the intestinal mucosal damage was scored according to the criteria described by Chiu et al. (1970), and microscopic scoring was performed blindly by two senior pathologists.

Immunohistochemistry was performed on serial sections of paraffin-embedded ileal tissue. After rehydrating, the sections were maintained in 0.3% H_2_O_2_ for 7 min to eliminate endogenous peroxidase and then were washed with phosphate buffer saline (PBS). Next, the samples were transferred to citrate buffer (pH 7.6) and heated in a microwave oven for 20 min. After washing the sections with PBS and blocking the non-specific binding sites with 5% bovine serum albumin (BSA), they were incubated with rabbit anti-cleaved caspase-3 (1:400), anti-Occludin (1:200), anti-Claudin 1 (1:100) polyclonal antibody overnight at 4°C. Next, the sections were rinsed in PBS and then incubated with biotin-labeled goat anti-polyvalent for 15 min at 37°C and horseradish peroxidase-labeled streptavidin for 20 min at 37°C. The coloration was completed after treatment with diaminobenzidine for 10 min, after which the slides were counterstained with hematoxylin for 2 min, rinsed in tap water and dehydrated. The sections were observed under a microscope. Based on the criteria proposed by Chen and Lin (2015), immunohistochemical staining was analyzed by two pathologists in a blind manner.

### Enzyme-Linked Immunosorbent Assay

The albumin, TNF-α, LPS, PCT, and CRP levels were determined using ELISA kits according to the manufacturer’s instructions. Briefly, 100 μl samples were added to each well of a 96-well plate that was precoated with a specific antibody, after which the plate was covered with sealer and incubated for 90 min at 37°C. After incubating with the biotinylated detection antibody working solution, 100 μl of the HRP-conjugated working solution was added to each well, and the plates were incubated for an additional 30 min at 37°C before adding 90 μl of substrate solution. The reaction was stopped by the addition of 50 μl of stop solution to each well. The absorbance of each well was read immediately at a wavelength of 450 nm, and the values were normalized to the control.

### Malondialdehyde Content Determination

The concentration of MDA, a reliable marker of lipid peroxidation, was determined using a thiobarbituric acid (TBA) assay kit according to the manufacturer’s instructions (Nanjing Jiancheng Bioengineering Institute, China). Briefly, ileal tissue samples were homogenized using a Retsch MM400 homogenizer (Retsch, Germany) followed by centrifugation at 3000 rpm/min for 15 min. Subsequently, the supernatants were collected for MDA measurements. MDA in the sample supernatant reacts with TBA at 95°C under acidic conditions, yielding a pink MDA-TBA conjugate. The optical density of the MDA-TBA complex at 532 nm was measured using a microplate reader (SpectraMax M5, United States). The total protein content in the ileal supernatant was analyzed using a Bradford Protein Assay kit (Tiangen Biotech Co., Ltd., Beijing, China). Finally, the MDA content (nanomoles per milligram protein) was calculated according to the formula described in the manufacturer’s instructions.

### Total Antioxidant Capacity Determination

The TAC was determined using a colorimetric method according to the manufacturer’s instructions (Nanjing Jiancheng Bioengineering Institute, China). The methods used for sample preparation and protein concentration determination were the same as those described in the previous section. Ferric tripyridyltriazine (Fe^3+^-TPTZ) is reduced to blue ferrous tripyridyltriazine (Fe^2+^-TPTZ) by various antioxidant components in the sample supernatant under acidic conditions. The absorbance of the blue product was measured at 520 nm using a microplate reader (SpectraMax M5, United States). Finally, the TAC (unit per milligram protein) was calculated according to the formula described in the manufacturer’s instructions.

### Serum Alanine Aminotransferase, Total Bilirubin, Triglyceride, and Hydroxyproline Analysis

Blood samples without anticoagulant from the inferior vena cava were centrifuged at 3,000 rpm for 15 min to collect the serum. The serum ALT, TBIL, triglyceride and hydroxyproline were determined colorimetrically with commercial assay kits according to the manufacturer’s protocols.

### Western Blot Analyses

Total protein was prepared using radioimmunoprecipitation assay buffer supplemented with 1× protease inhibitor. The total protein content in the ileal supernatant was analyzed using a Bradford Protein Assay kit (Tiangen Biotech Co., Ltd., Beijing, China). The protein samples were loaded (30 μg/well) and separated using an SDS-polyacrylamide gel. The gel was transferred to a nitrocellulose membrane and blocked with 5% skim milk in Tris–buffered saline with Tween 20 (TBST). Next, the membrane was incubated with specific primary antibodies overnight at 4°C followed by incubation with horseradish peroxidase-conjugated secondary antibodies for 4 h at 4°C. The membrane was treated with chemiluminescence reagent and exposed to a luminescence image analyzer (Bio-Rad ChemiDoc MP, United States) to detect the protein bands. The relative levels of the target protein were expressed as a gray intensity ratio of the target band to the β-actin band.

### 16S Ribosomal RNA Gene Sequencing

The V3–V4 region of the bacterial 16S ribosomal RNA (rRNA) gene was PCR amplified with indexed primers (338F and 806R) using FastPfu Polymerase. The amplicons were then purified by gel extraction and quantified using a QuantiFluor-STusing E.Z.N.A. Soil DNA Isolation kit. The purified PCR products were used for high-throughput pyrosequencing, which was carried out by Majorbio Bio-Pharm Biotechnology Co., Ltd., Shanghai, China, using an Illumina MiSeq PE250. The resulting sequences were analyzed using Quantitative Insights into Microbial Ecology. All the sequences were clustered into operational taxonomic units (OTUs) based on a 97% identity threshold by the SILVA database. A representative sequence from each OTU was selected for downstream analysis, and the community richness and diversity indices were calculated.

### Statistical Analysis

Quantitative data were expressed as the means ± standard deviation (SD). Normality was assessed using the single-sample Kolmogorov-Smirnov Test, and normally distributed data were analyzed by one-way analysis of variance (ANOVA) followed by the least significant difference test. Ranked data were compared by the Kruskal-Wallis *H*-test among the groups. If positive, multiple comparisons were carried out using the Nemenyi test. Statistical analyses were performed with IBM SPSS statistics version 23.0. Values of *P* < 0.05 were considered significant.

## Results

### UA Suppresses Hepatic Fibrogenesis in Rats With CCl4-Induced Liver Fibrosis

**FIGURE 1:**
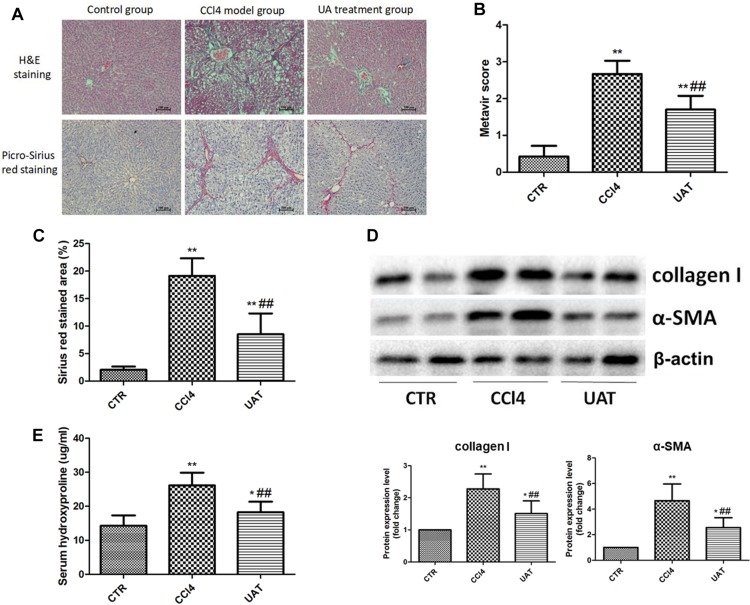
Ursolic Acid ameliorates liver injury and suppresses hepatic fibrogenesis in rats with CCl4-induced liver fibrosis. **(A)** Liver sections were stained with H&E and Picro-Sirius red, and representative images are shown. **(B)** Morphological changes in the liver sections quantified using the Metavir score. **(C)** The Sirius red stained area was quantified by Image-Pro Plus 6.0. **(D)** The protein levels of collagen I and α-SMA in liver tissues were analyzed by Western blot assays. **(E)** The level of hydroxyproline was measured by chemical oxidation. Original magnification: 100×. The data are presented as the means ± SD (*n* = 8 per group). ^*^P < 0.05; ^∗∗^*P* < 0.01 versus the control group and ^##^*P* < 0.01 versus the CCl4 model group.

To investigate the effects of UA on liver fibrosis, a rat model of CCl4-induced liver fibrosis was established. During the modeling, the mortality rate for the control group was 0% (0/10), while that of the CCl4 model and UA treatment groups was 20% (2/10). As shown in Figure 1A, tissue sections from the control group showed a normal hepatic lobular structure and little collagen deposition. Heavy deposits of collagen were observed in the livers of rats from the CCl4 model group and were accompanied by disordered hepatic lobular structures and severe hepatocyte necrosis, whereas these changes were suppressed in the UA treatment group. In addition, the hepatic fibrosis scores (Figure 1B) and area (Figure 1C) in the UA treatment group were significantly lower than those of the CCl4 model group. The expression of collagen I and α-SMA were significantly elevated in the CCl4 model group compared with that observed in the control group, which was significantly inhibited by the UA treatment (Figure 1D). The hydroxyproline content of rat serum in the UA treatment group declined compared to the CCl4 model group (Figure 1E). These results confirmed that UA protected the rat liver from fibrogenesis induced by CCl4.

### UA Ameliorates Liver Injury in Rats With CCl4-Induced Liver Fibrosis

**FIGURE 2:**
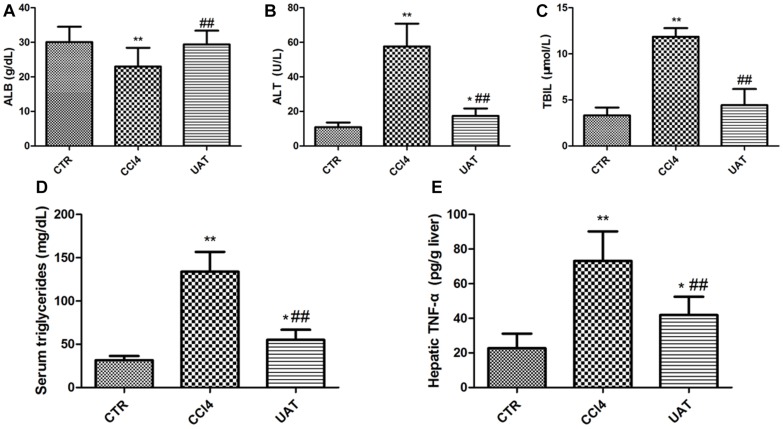
UA ameliorates Liver Injury in Rats with CCl4-Induced Liver Fibrosis. **(A)** The serum albumin was determined using the ELISA kit. The serum ALT **(B)**, TBIL. **(C)** and triglyceride **(D)** were determined colorimetrically with commercial assay kits. **(E)** The hepatic TNF-α was determined using the ELISA kit. The data are presented as the means ± SD (*n* = 8 per group). ^*^P < 0.05 versus the control group; ^∗∗^*P* < 0.01 versus the control group; and ^##^*P* < 0.01 versus the CCl4 model group.

We measured the contents of ALT, ALB, TBIL, and triglyceride in rat’s serum. The results presented in Figures 2A–D showed decreased serum ALB and increased serum ALT, TBIL and triglyceride in the CCl4 model group compared to those in the control group, which were partly restored by UA. As shown in Figure 2E, the hepatic TNF-α level was significantly higher in CCl4 model group than in the control group. However, the increased level of hepatic TNF-α was reduced by UA treatment. Additionally, the UA treatment group had higher final body weights and lower liver weights than the CCl4 model group (Table 1).

**TABLE 1:** Characteristics of rats in control, CCl4 model and UA treatment groups.

**Indexes**	**Control group**	**CCl4 model group**	**UA treatment group**
	**(*n* = 10)**	**(*n* = 8)**	**(*n* = 8)**
Initial body weight (*g*)	190.31 ± 5.23	192.74 ± 4.85	192.45 ± 4.32
Final body weight (*g*)	452.52 ± 20.33	368.65 ± 32.56^**^	415.09 ± 24.86^*^^#^
Body weight increase (*g*)	262.11 ± 10.85	175.93 ± 20.61^**^	223.26 ± 17.25^*^^#^
Liver weight (*g*)	135.74 ± 4.60	162.20 ± 10.96^**^	155.64 ± 8.74^**^
Liver weight/Final body weight	0.30 ± 0.01	0.44 ± 0.03^**^	0.38 ± 0.06^*^^#^
Albumin in feces (ng/mg)	15.11 ± 5.80	36.40 ± 10.01^*^	21.33 ± 8.12^*^^#^

The data are presented as the means ± SD. ^*^P < 0.05 versus the control group; ^∗∗^*P* < 0.01 versus the control group; ^#^*P* < 0.01 versus the CCl4 model group.

### UA Improves Intestinal Dysbiosis in Rats With Liver Fibrosis

**FIGURE 3:**
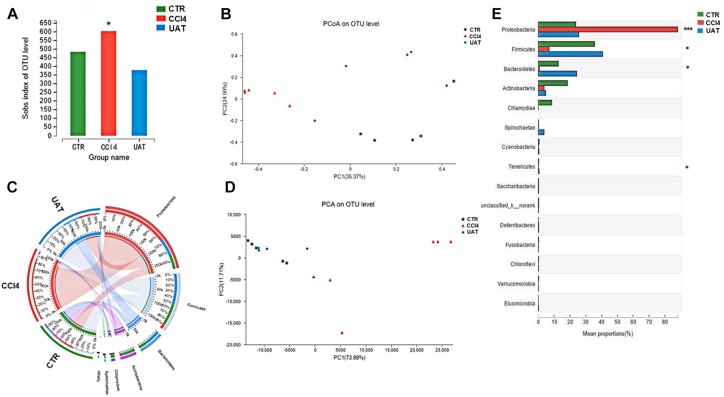
Bacterial 16S rRNA gene sequencing of the ileal mucosa of rats with liver fibrosis (*n* = 5). **(A)** Analysis of α-diversity. The horizontal coordinate is the sample name and the vertical coordinate is the diversity index of the selected classification level. Intestinal bacterial α-diversity, indicated by the number of observed OTUs, was reduced in UA treatment groups (*p* = 0.045, Wilcoxon rank-sum test). **(B)** The results of the PCoA. Different color points represent samples from different groups, and the spatial distance of the sample points represents the differences between the samples. PCoA of unweighted UniFrac analysis demonstrated that the UA treatment group was significantly different from the CCl4 model group (pseudo-F: 3.61, *p* = 0.001, PERMANOVAR). **(C)** LEfSe analysis. Significant differences in the abundances of specific taxa were observed among the groups. The estimated effect of the abundance of each group was estimated. **(D)** PCA demonstrated that the intestinal bacterial communities of the UA treatment and CCl4 model groups could be separated using at the phylum level. **(E)** Analysis of significant differences between the groups. The vertical coordinate represents the species names at different classification levels, the horizontal coordinate represents the relative abundance of a species in a sample, and different colors represent different groups. (^*^P < 0.05 and ^∗∗∗^*P* < 0.001 compared to the control group).

We assessed the abundance and diversity of microbes in the ileal mucosa of 5 randomly selected rats from each group by analyzing the 16S rRNA gene sequences of collected ileal tissue samples. The results presented in Figure 3 showed that the abundance of intestinal flora, as measured by numbers of observed OTUs, was reduced in the UA treatment group compared to that observed in the CCl4 model group (*p* = 0.038, Nemenyi test; Figure 3A). However, the Shannon index, which measures both richness and evenness, was not significantly different between the CCl4 model and UA treatment groups (*p* = 0.40). An unweighted UniFrac-based principal coordinates analysis (PCoA) revealed that the overall microbial composition of the UA treatment group deviated from the CCl4 model group (PERMANOVAR, pseudo-F: 3.61, *p* = 0.001, Figure 3B). Principle component analysis (PCA) demonstrated that the intestinal bacterial communities of the three groups could be separated at the phylum abundance level (Figure 3D). LEfSe analysis (Figure 3C) and analysis of significant differences between the groups (Figure 3E) were performed to evaluate the relationships between the UA treatment and CCl4 model groups. Significant increases in the abundances of some bacterial phyla were observed in the UA treatment group, including Proteobacteria (*p* = 0.000), Firmicutes (*p* = 0.012), Actinobacteria (*p* = 0.011) and Tenericutes (*p* = 0.033). In general, the genera enriched in the CCl4 model group should be indirectly correlated with those enriched in the UA treatment group (Figure 3), suggesting an antagonistic relationship between harmful and beneficial bacteria.

### UA Ameliorates Intestinal Mucosal Barrier Injury and Systemic Inflammation in Rats With CCl4-Induced Liver Fibrosis

**FIGURE 4:**
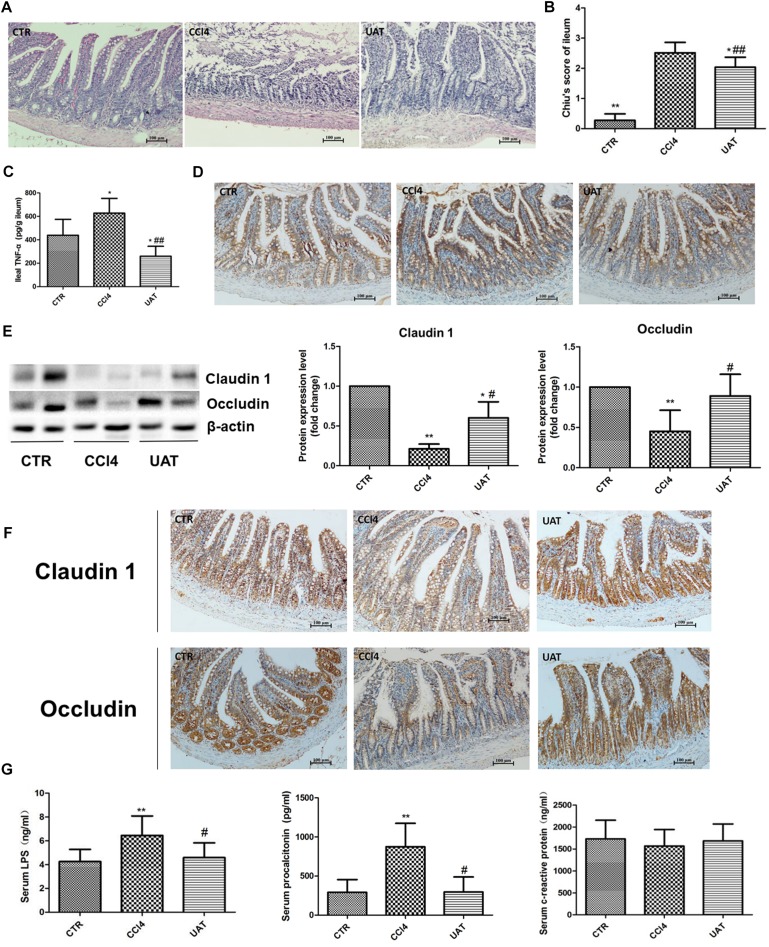
UA ameliorates intestinal mucosal barrier injury and systemic inflammation in rats with CCl4-induced liver fibrosis. **(A)** Ileal sections were stained with H&E, and representative images are shown. **(B)** Morphological changes in the ileal sections quantified using Chiu’s score. Original magnification: 100×. **(C)** The ileal TNF-α was measured by ELISA. **(D)** Immunohistochemical staining of cleaved caspase-3 in ileal epithelial cells, with representative images shown. **(E)** The levels of the tight junction proteins Claudin 1 and Occludin in ileal tissues were analyzed by Western blot assays. **(F)** Immunohistochemical staining of Claudin 1 and Occludin in ileal tissue, with representative images shown. **(G)** The levels of serum LPS, procalcitonin, and CRP were measured by ELISA. The data are presented as the means ± SD (*n* = 8 per group). ^*^P < 0.05 versus the control group; ^∗∗^*P* < 0.01 versus the control group; ^#^*P* < 0.01 versus the CCl4 model group; and ^##^*P* < 0.01 versus the CCl4 model group.

We evaluated the pathological changes in the ileal tissue, and the results presented in Figure 4A showed that the tissue sections from rats in the control group exhibited normal intestinal structures and intact mucosa. In contrast, the ileal sections from the CCl4 model group showed disturbances in mucosal structure, including villous edema, atrophy, exfoliation and focal inflammatory cell infiltration in the lamina propria as well as mild desquamation of the mucosal epithelium lining in some areas, which was partly restored by UA treatment. Additionally, the UA treatment group had lower Chiu scores than the CCl4 model group (Figure 4B). The ileal TNF-α level was significantly higher in CCl4 model group than in the control group. However, the increased level of ileal TNF-α was reduced by UA treatment (Figure 4C).

We next assessed the expression of the apoptosis protein caspase-3 in ileal epithelial cells. The results presented in Figure 4D and Table 2 showed that elevated cleaved caspase-3 immunoreactivity was detected in the CCl4 model group compared to that in the control group, which was reduced by UA treatment.

**TABLE 2:** Immunohistochemical score for cleaved caspase 3 in ileal epithelial cells.

**Group**	**N**	**Cleaved caspase 3 expression intensity**			
		**−**	**+**	**++**	**+++**
Control group	10	9	1	0	0
CCl4 model group^∗∗^	8	0	1	2	5
UA treatment group^#^	8	3	4	1	0

*^∗∗^*P* < 0.01 versus the control group; ^#^*P* < 0.01 versus the CCl4 model group.*

Tight junction proteins play an important role in the maintenance of intestinal barrier integrity and permeability. Therefore, we examined the expression of the tight junction proteins Claudin 1 and Occludin in ileal tissues. As shown in Figures 4E,F and Tables 3, 4, the expressions of the tight junction proteins Claudin 1 and Occludin were significantly lower in the CCl4 model group than in the control group. However, the reduced ileal expressions of Claudin 1 and Occludin were restored by UA. Besides, the feces albumin content in the UA treatment group was significantly lower than that observed in the CCl4 model group (Table 1).

**TABLE 3:** Immunohistochemical score for Claudin 1 in ileal tissue.

**Group**	**N**	**Claudin 1 expression intensity**			
		**−**	**+**	**++**	**+++**
Control group	10	0	0	2	8
CCl4 model group^∗∗^	8	4	4	0	0
UA treatment group^#^	8	0	1	5	2

*^∗∗^*P* < 0.01 versus the control group; ^#^*P* < 0.01 versus the CCl4 model group.*

**TABLE 4:** Immunohistochemical score for Occludin in ileal tissue.

**Group**	**N**	**Occludin expression intensity**			
		**−**	**+**	**++**	**+++**
Control group	10	0	0	1	9
CCl4 model group^∗∗^	8	3	4	1	0
UA treatment group^#^	8	0	1	4	3

*^∗∗^*P* < 0.01 versus the control group; ^#^*P* < 0.01 versus the CCl4 model group.*

Intestinal barrier injury contributes to bacterial translocation and systemic inflammation, which we assessed by measuring serum LPS, PCT and CRP levels. Compared with those in the control group, increased serum LPS and PCT levels were observed in the CCl4 model group, while serum CRP levels showed no significant changes. The serum LPS and PCT levels in the UA treatment group were lower than those observed in the CCl4 model group. In addition, serum the CRP levels showed no significant differences between the UA treatment and CCl4 model groups (Figure 4G).

### UA Inhibits Intestinal Oxidative Stress Mediated by NADPH Oxidase in Rats With CCl4-Induced Liver Fibrosis

**FIGURE 5:**
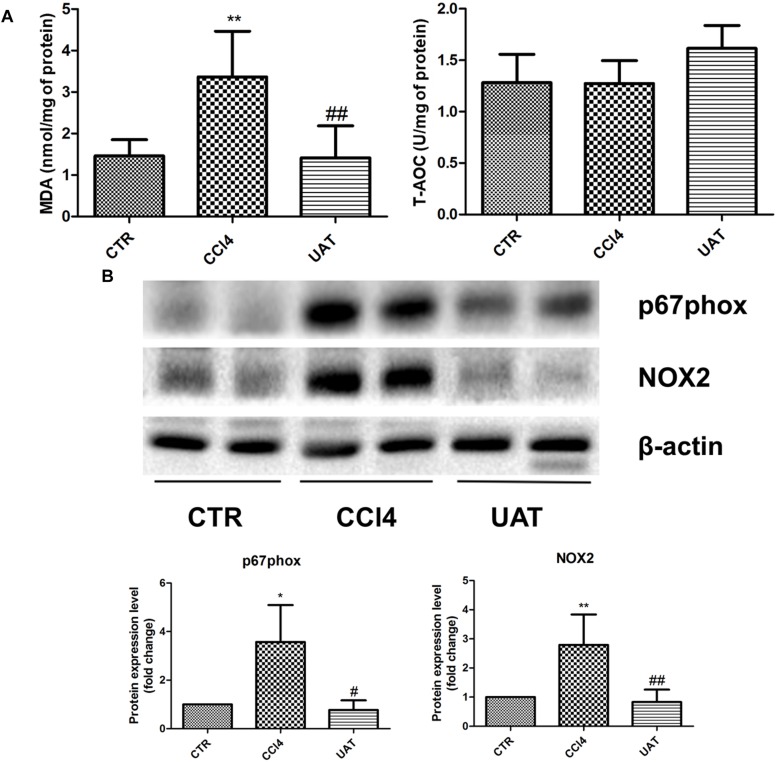
UA inhibits intestinal oxidative stress mediated by NADPH oxidase in rats with CCl4-induced liver fibrosis. **(A)** The ileal malondialdehyde (MDA) levels and total antioxidant capacity (TAC) were measured using thiobarbituric acid (TBA) and colorimetric methods, respectively. **(B)** The protein levels of P67phox and NOX2 in ileal tissues were analyzed by Western blot assays. The data are presented as the means ± SD (*n* = 8 per group). ^*^P < 0.05 versus the control group; ^∗∗^*P* < 0.01 versus the control group; ^#^*P* < 0.01 versus the CCl4 model group; and ^##^*P* < 0.01 versus the CCl4 model group.

To elucidate the mechanisms by which UA improves the intestinal mucosal barrier, we investigated the impact of UA on MDA levels and the TAC in the ileum of rats. Increased ileal MDA levels were observed in the CCl4 model group compared with those observed in the control group, whereas no significant changes in TAC were observed. The ileal MDA contents decreased in response to the UA treatment, and no significant difference in the TAC was observed between the UA treatment and CCl4 model groups (Figure 5A).

We next assessed the expression of the NOX protein components P67phox and NOX2 in ileal tissues by Western blot assays. The results presented in Figure 5B showed that P67phox and NOX2 expression was significantly elevated in the CCl4 model group compared with that observed in the control group, which was significantly inhibited by the UA treatment.

## Discussion

Hepatic fibrosis is a common outcome of a variety of chronic liver diseases that is characterized by the accumulation of ECM, primarily type I collagen. The exact mechanism of liver fibrogenesis is still largely unknown, and a variety of factors contribute to fibrogenesis. The results of our previous study (Gan et al., 2018) showed that oxidative stress derived from NOX plays an important role in the pathogenesis of liver fibrosis and participates in regulating various signaling pathways involved in hepatic fibrosis. UA inhibited HSC activation by suppressing NOX activity and expression, preventing liver fibrosis (He et al., 2015). In the current study, we reevaluated the antifibrotic effects of UA, a natural pentacyclic triterpenoid carboxylic acid, using a classical animal model of liver fibrosis that causes hepatocellular necrosis and the deposition of collagen in the liver. Compared to the CCl4 model group, the fibrous septum and collagen deposition were reduced in the liver tissue of the UA treatment group. The serum ALT, TBIL, and triglyceride in the UA treatment group declined compared to the CCl4 model group. However, the serum albumin and final body weight were increased. All these results indicate that UA effectively improved liver histology and hepatocellular necrosis and inhibited collagen production in the livers of rats displaying damage and fibrogenesis caused by CCl4, which is consistent with the results of our previous studies showing that UA has unique antifibrotic effects.

The translocation of bacteria and their products across the intestinal barrier is common in liver disease, and there is evidence that experimental liver fibrosis depends on bacterial translocation (Mazagova et al., 2015). Dysbiosis can cause intestinal inflammation, disruption of the gut barrier, and bacterial translocation. Subsequently, translocated bacterial products induce hepatic inflammation, liver damage, liver fibrosis or even liver cirrhosis (Schnabl and Brenner, 2014). Fouts et al. (2012) confirmed that liver fibrosis is associated with an increase in adherent aerobic and anaerobic bacteria in the small and large intestine in a rat model of liver fibrosis induced with intraperitoneal injections of CCl4, where CCl4-induced liver injury was accompanied by intestinal bacterial overgrowth and dysbiosis. The distinctive pattern of dysbiosis observed in our model is similar to that described by Ubeda et al. (2016) in that cirrhotic rats displayed a reduced number of Firmicutes OTUs and an increased number of Proteobacterial OTUs. Intestinal dysbiosis was partly resolved in rats with liver fibrosis in response to the UA treatment, which restored the relative abundances of Proteobacteria and Firmicutes to that of the control mice. Although the composition of microbiome changed by CCl4 administration was restored by UA, it is still unknown whether intestinal dysbiosis is directly associated with liver injury and fibrosis in the present study. The cause of intestinal dysbiosis likely includes an absence or decrease in the intestinal levels of bile acids, changes in intestinal motility or feeding rates, etc. There is evidence that conjugated bile acids promote innate defense against luminal bacteria by regulating the expression of host genes (Inagaki et al., 2006). Through a similar mechanism, UA can activate the intracellular killing activity of macrophages against bacteria during infections (Podder et al., 2015). In addition, UA possesses direct antibacterial activity (Grace et al., 2016). All these factors may contribute to the effect of UA toward intestinal flora, which requires further study.

The intestinal mucosal barrier is an important modulator of intestinal homeostasis that consists of a permeable monolayer of epithelial cells. The epithelium allows for the absorption of nutrients while providing a physical barrier to prevent the translocation of potentially harmful substances, including pathogens, toxins, and antigens, from the gut lumen into the mucosal tissues and circulatory system via transcellular and paracellular pathways. The transcellular pathway is predominantly mediated by specific transporters or channels located on the apical and basolateral membranes (Suzuki, 2013). The paracellular pathway is regulated by apical junctional complexes, consisting of tight junctions, adherent junctions and desmosomes (Odenwald and Turner, 2017). Tight junctions, the primary determinants of paracellular permeability, seal the paracellular space and form a barrier that exhibits both size and charge selectivity with two distinct routes, termed the “pore” and “leak” pathways. The pore pathway refers to a high-capacity, size-selective and charge-selective route that appears to be primarily regulated by claudins, whereas the leak pathway is a low-capacity pathway that is regulated by Occludin (Odenwald and Turner, 2017). In the present study, the ileum of rats in the CCl4 model group showed complicated mucosal structures, villous atrophy, and increased enterocyte apoptosis, which were significantly improved following UA treatment. Moreover, the results of Western blot and Immunohistochemical assays demonstrated that the expressions of the tight junction proteins Claudin 1 and Occludin were effectively upregulated in the UA treatment group compared with that in the CCl4 model group. The feces albumin content in the UA treatment group was significantly lower than that observed in the CCl4 model group. All these results indicated that an impaired intestinal mucosal barrier was present in rats with liver fibrosis induced by CCl4 and that the UA treatment could effectively ameliorate intestinal mucosal barrier injury.

An impaired intestinal mucosal barrier, including decreased expression of tight junction protein, results in increased intestinal permeability, which contributes to the translocation of bacteria and/or bacterial products (LPS, bacterial DNA, etc.) (Fouts et al., 2012; De Minicis et al., 2014). Translocated bacteria and/or bacterial products lead to intestinal endotoxemia and trigger systemic inflammatory responses. In the present study, intestinal endotoxin and a systemic inflammatory response were observed in rats with CCl4-induced liver fibrosis, as demonstrated by the increased serum LPS and procalcitonin in the CCl4 model group. However, no significant differences in the serum CRP levels were observed between the CCl4 model group and control groups. This result likely occurred because CRP is less sensitive than procalcitonin in response to systemic inflammation. The increased levels of serum LPS and procalcitonin were abolished in the UA treatment group, suggesting that UA can ameliorate intestinal endotoxemia and systemic inflammatory responses. Intestinal barrier injury promotes intestinal endotoxemia and systemic inflammatory responses. After UA treatment, we observed an improved mucosal structure, decreased inflammatory cell infiltration in the lamina propria and increased expression of tight junction proteins in the ileum of rats. These results indicated that UA reduced the serum LPS and procalcitonin contents by ameliorating the intestinal mucosal barrier injury in rats with CCl4-induced liver fibrosis.

Compromised intestinal barrier function has been shown to be associated with numerous disease states, both intestinal and systemic. Various factors contribute to intestinal barrier damage in a pathological state, especially intestinal oxidative stress. Oxidative stress promotes intestinal barrier dysfunction through a variety of mechanisms and can impair the epithelial barrier by directly oxidizing cell components and inducing cell apoptosis. ROS impacts epithelial cells by altering mucosal glycosylation and increasing bacterial adherence, internalization and translocation (Natarajan et al., 2006; Schoultz et al., 2012). MDA is produced as a result of ROS formation from the oxidation of membrane lipids and is commonly used as a marker to indicate the level of oxidative damage in tissues (Chiva et al., 2003). In our present study, oxidative stress was observed to contribute to intestinal barrier damage as indicated by the elevated MDA levels in the intestinal tissues from the CCl4 model group. Accompanied by the decreased MDA levels, an amelioration of intestinal barrier dysfunction was observed in the UA treatment group, demonstrating that UA protected the intestinal mucosal barrier by inhibiting intestinal oxidative stress.

The antioxidant system consists of enzymatic and non-enzymatic components. The enzymatic antioxidant components include superoxide dismutase, glutathione peroxidase, catalase, etc., whereas the non-enzymatic antioxidant components include vitamins, amino acids, and metalloproteins, etc. Our results showed that ileal TAC displayed no significant differences between the control and CCl4 model groups. There are two possibilities for these outcomes: either the intestinal antioxidant system is not affected in rats with CCl4-induced liver fibrosis or the antioxidant capacity of some components of the antioxidant system is destroyed and is compensated for by other components.

Several differentially localized and expressed enzymatic systems contribute to ROS formation in the gut, including the mitochondrial respiratory chain, xanthine oxidase, nitric oxide synthase, NOX, etc. (Bhattacharyya et al., 2014). NOX is a multimeric transmembrane enzyme complex that generates ROS in response to diverse stimuli. The classical phagocytic NOX consists of a heterodimeric membrane-bound flavocytochrome b558 complex, the catalytic subunit gp91phox (renamed NOX2) and the regulatory subunit p22phox located in the membrane, as well as the cytoplasmic regulatory components P67phox, p47phox, p40phox, and Rac1 (Paik et al., 2014). Intestinal NOX-mediated oxidative stress is closely associated with intestinal mucosal barrier damage in a variety of pathological conditions. Xie et al. (2014) observed that oxidation protein products trigger intestinal epithelial cell death and intestinal tissue injury via a NOX-mediated redox signaling pathway in Crohn’s disease. The NOX inhibitor apocynin reduces intestinal mucosal barrier injury in a rat model of severe acute pancreatitis (Deng et al., 2016). The Western blot results from the present study showed that the expression of intestinal P67phox and NOX2 was higher in the CCl4 model group than in the UA treatment group. Moreover, MDA levels were also higher in the CCl4 model group. Considering the amelioration of intestinal barrier dysfunction observed in the UA treatment group, these results support that UA protects the intestinal mucosal barrier in rats with hepatic fibrosis by inhibiting the expression of intestinal NOX and oxidative stress derived from NOX.

In summary, the results of the present study demonstrated that UA has protective effects on the intestinal mucosal barrier in rats with CCl4-induced liver fibrosis by inhibiting intestinal NOX-mediated oxidative stress. Given the positive effect of UA on the intestinal mucosal barrier, we anticipate that these findings could be a stepping stone for developing UA as a novel antifibrotic agent.

## Ethics Statement

This study was carried out in accordance with the recommendations of the humane care in compliance with institutional guidelines, Institutional Animal Care and Use Committee of the First Affiliated Hospital of Nanchang University. The protocol was approved by the Institutional Animal Care and Use Committee of the First Affiliated Hospital of Nanchang University.

## Author Contributions

WZ, DG, and JJ contributed equally to this study. WZ was responsible for experiments and manuscript writing. DG was responsible for the project design. FL and SW were responsible for molecular biology experiments. CH and MJ were responsible for the cell slide and color rendering, grading, etc. YW, AW, and BL were responsible for assisting in the data processing and picture modification. XZ was responsible for the final modification of the manuscript. WZ and DG conducted the experiments, and planned and wrote the manuscript. JJ conducted the revision of the manuscript. CH, FL, SW, MJ, and YW conducted the experiments and data analysis. AW helped to perform the experiments and wrote the manuscript. BL and XZ collaborated with the other authors to correct the manuscript.

## Conflict of Interest Statement

The authors declare that the research was conducted in the absence of any commercial or financial relationships that could be construed as a potential conflict of interest.
